# “Being Brave on Social Media”: Coping with Bias-Based Cyberaggression Among Racialized Youth

**DOI:** 10.3390/bs16101882

**Published:** 2026-10-13

**Authors:** Johanna Sam, Haoyu Zhao, Daniel Gallardo, Sabrina Forcier, Claire Hein-Salvi, Rachel Hussey, Jennifer Shapka, Jan Hare

**Affiliations:** 1 Faculty of Education, The University of British Columbia, Vancouver, BC V6T 1Z4, Canada; 2 Faculty of Behavioural Sciences, Yorkville University, Fredericton, NB E3C 2R9, Canada

**Keywords:** bias-based cyberaggression, adolescence, social media, resilience, coping strategies, mixed-methods research

## Abstract

Bias-based cyberaggression refers to hurtful online actions that target an individual based on socio-demographic characteristics (e.g., ethnicity or race). Empathy tends to be lower among cyberaggression perpetrators. The study used a mixed-methods design, including questionnaire filling-out, open-text responding, and collaborative online empathy mapping, to explore cyberaggression experiences among racialized youth. Thirteen youths (aged 13 to 25) joined the present study. Participants were mostly heterosexual young men (*n* = 11) who self-identified as Black (*n* = 12) and Indigenous (*n* = 1). Participants resided in Canada (*n* = 4), the USA (*n* = 4), Kenya (*n* = 4), and South Africa (*n* = 1). Findings from quantitative data suggest that participants perpetrated bias-based cyberaggression behaviors, targeting others’ sexual orientations and gender identities; they were also victims of cyberaggression for their racial identity. Qualitative findings describe targeted cyberaggression, hate speech, and hacking of social media profiles. Peers encourage them to perpetrate cyberaggression and think about how they “[want to] settle the score.” Instead, participants did not respond to hurtful posts. Participants did seek social support to cope with cyberaggression from their siblings, close relatives, and friends. Findings indicate that for research on online wellbeing, it is important to engage diverse youth with lived experience of cyberaggression. The results provide preliminary support for empathy as a potential focus of bias-based cyberaggression prevention and intervention efforts. Future research should explore these relationships in larger, more diverse samples and examine empathy-based interventions to promote safer online environments.

## 1. Introduction

Substantial screen time and internet access have led to many issues among youth. Among these device-use issues, cyberaggression is a prominent concern that has drawn widespread public health attention. To raise awareness of this maladaptive technology engagement, the United Nations Educational, Scientific, and Cultural Organization (UNESCO) announced in 2019 that the first Thursday of November would be the International Day against Violence and Bullying at School, including cyberaggression. Since then, the past several years during the COVID-19 pandemic have brought rapid and dramatic changes in youth’s lives, especially due to prolonged closures of face-to-face spaces and long-lasting social isolation. This increased youth’s use of online platforms and mobile devices to stay socially connected during the pandemic, which may have increased their risk of experiencing cyberaggression. In this regard, systematic review findings indicate youth’s cyberaggression roles and prevalence during the COVID-19 pandemic ranged from 2% to 12% involved in perpetrating cyberaggression (i.e., cyberaggressing others); 8% to 49% had experienced cybervictimization (i.e., being cyberaggressed); and lastly, 2% to 10% reported a dual role of cyberaggression perpetrators and cybervictims (Sorrentino et al., 2023). Similar prevalence rates among youth were also reported after the pandemic (e.g., Chen, 2024; Hinduja & Patchin, 2025; Ma et al., 2024; Pérez-Albéniz et al., 2025). Given the lack of a universal definition, systematic reviews often found variation in reported rates of cyberaggression across countries (Henares-Montiel et al., 2022). Thus, the current understanding of cyberaggression, referred to as “using information and communication technologies to repeatedly and intentionally harm, harass, hurt, and/or embarrass a target” based on a recent concept analysis (Peter & Petermann, 2018), may not fully consider the lived experiences of diverse youth (Kofoed & Staksrud, 2019). For instance, past research has found ethnic differences in U.S. youth’s online practices and experiences (Pew Research Center, 2025). Overall, nearly 96% of youth go online daily, and 46% of them are online almost all the time; however, Hispanic youth and Black youth exhibited higher proportions of being online almost all the time, with 58% and 53%, respectively; in comparison, only 37% of White youth report going online almost all the time (Pew Research Center, 2025). In regard to technology use differences, past study findings indicate Asian and multi-racial youth checked their social media sites less frequently in comparison to other racialized and White youth (Hernandez et al., 2023). Race and ethnicity are part of youth intersectional identities, which are now dynamically developing online (Hernandez et al., 2023). Yet, bias-based cyberaggression has not been widely explored.

Bias-based cyberaggression refers to hurtful online actions that target an individual based on actual or perceived sociodemographic characteristics (e.g., sexual orientation, gender, ethnicity, religion; Hinduja & Patchin, 2022). It is a subtype of the broader concept of cyberaggression and was central to the present study. For instance, youth who were sexual and gender minorities have reported more bias-based cybervictimization experiences than their heterosexual and cisgender counterparts (Sherwood et al., 2025). Specifically, for bias-based cyberaggression that targets race and ethnicity, a previous study has shown that compared with the majority group, immigrant and second-generation youth had substantially higher rates of bias-based cybervictimization (Zych & Llorent, 2023). Online racism (e.g., discrimination against Black and Latino youth) was also reported and was found to be associated with poor mental health (Polanco-Roman et al., 2025; Tynes et al., 2024, 2025). These bias-based cyberaggression experiences may place racialized youth at greater risk of adverse outcomes because they involve identity-based harm that extends beyond general cyberaggression. Exploring how racialized youth navigate and cope with these experiences is necessary to better understand protective processes and inform targeted supports.

According to the Transactional Model of Stress and Coping proposed by Lazarus and Folkman (1984), individuals’ responses to cyberaggression experiences may be shaped by how they appraise the event and the *coping strategies* available to them. *Primary appraisal* involves evaluating the significance of a stressful experience. In contrast, *secondary appraisal* involves assessing whether available resources or actions can manage the stressor (Lazarus & Folkman, 1984). For example, a primary appraisal of a cyberaggression incident may involve determining whether a social media post was hurtful, whereas the secondary appraisal may involve considering whether one has access to a supportive social network or other resources to respond. From this perspective, coping strategies refer to the cognitive and behavioral efforts individuals use to manage stressful experiences, and youth’s appraisals of online stressors may influence coping strategies they employ.

Coping strategies are conceptually categorized into four dimensions, notably active, distraction, avoidance, and support seeking (Ayers et al., 1991, 1996). *Active coping strategies* may include decision-making, positive thinking, minimization, and/or seeking understanding of problematic circumstances. *Distraction coping strategies* may involve efforts to physically release emotions (e.g., playing sports, exercise) and/or distracting actions (e.g., hobbies, video games, or entertainment). *Avoidance coping strategies* may involve avoidant actions (e.g., staying away from upsetting things), repression (e.g., not thinking about it), and/or wishful thinking. *Support-seeking coping strategies* are distinguished by support for actions through others to seek solutions, advice, or information, as well as support for feelings. This involves others in listening to feelings or providing understanding to help the person feel less upset. Appraisal-based transactional processes can lead youth to adopt active or supportive-seeking coping strategies that focus on problem-solving, or to initiate distraction or avoidance coping strategies that are emotion-based (Lazarus & Folkman, 1984). Youth with protective factors, such as higher school connectedness or more prosocial behaviors, were more likely to use problem-focused rather than emotion-focused coping strategies (Chan & Wong, 2017).

Racialized youth who have experienced bias-based cyberaggression were at increased risk of psychological maladjustment and substance misuse (Keum et al., 2023). Yet, not all youth who experienced cyberaggression had similar psychological consequences, and coping strategies might play an essential role (Erbiçer et al., 2023; Quintana-Orts et al., 2023; Yang et al., 2024). For instance, past study findings showed that a maladaptive coping strategy of blaming others played the most salient role for predicting cyberaggression perpetration and cybervictimization among youth (Quintana-Orts et al., 2023). Within cyberaggression research, emerging evidence suggests that youth’s coping responses influence how they navigate online harms. Research on cyberhate has identified constructive coping strategies, including assertiveness, technical coping, and close support, as common responses to cyberhate experiences, with greater use of adaptive coping strategies associated with better youth wellbeing (Gámez-Guadix et al., 2020; Wachs et al., 2019). Despite growing interest in the role coping strategies play in cyberaggression, little is known about what types of coping strategies are utilized by racialized youth in response to cyberaggression experiences. While the literature has focused on coping strategies in response to cyberaggression (Arató et al., 2020), less attention has been paid to racialized youth’s online experiences and the particular coping strategies they employed.

Related to coping strategies, *resilience* refers to a dynamic process between youth and their environments to positively adapt in the face of adversity or stressful events (Naglieri et al., 2013; Wagnild & Young, 1993). Some scholars have claimed that resilience is an inferential concept that involves two judgments: (i) that an individual has experienced risk or adversity, and (ii) that the individual positively adapts despite this risk exposure (M. O. Wright et al., 2013). The Social Ecology of Resilience Framework has raised concerns that most resilience research is conducted from a Western scientific perspective and lacks assessments that account for diverse cultural and contextual backgrounds (Ungar, 2008, 2011). According to Ungar (2008, p. 225), resilience should be defined under an ecological framework as “exposure to significant adversity; resilience is both the capacity of individuals to navigate their way to the psychological, social, cultural, and physical resources that sustain their wellbeing, and their capacity individually and collectively to negotiate for these resources to be provided and experienced in a culturally meaningful way”. Indeed, although past study findings have revealed that youth with high levels of resilience had a lower risk of experiencing cyberaggression and suffering from fewer adverse effects (Hinduja & Patchin, 2017; Korkmaz et al., 2025; Santos et al., 2021), evidence remains limited regarding how resilience shapes youth experiences of cyberaggression across racialized and cultural contexts.

Given the culturally and contextually specific dimensions of resilience proposed by Ungar (2011), this study draws on a culturally rooted understanding of the “new warrior” approach to resilience (Alfred, 2005). Within Indigenous teachings, the concept of a warrior is not understood as aggression or conflict, but rather as a commitment to responsibility, courage, protection, and maintaining relationships with family, community, and future generations. As Louie (2021) articulates, the warrior identity is grounded in discipline, responsibility, and contributing to the strength and wellbeing of one’s community, rather than individual achievement or domination. This understanding reflects Indigenous perspectives of resilience as relational, where individuals draw upon cultural knowledge, collective strengths, and community relationships to navigate adversity. As described by Hare and Pidgeon (2011), Indigenous youth have drawn upon cultural identity and relational connections to respond to experiences of bullying and marginalization within school contexts. Building on these perspectives, the present study extends this culturally grounded understanding of resilience to online contexts by exploring how racialized youth navigate cyberaggression experiences. Specifically, the study affords youth a voice to describe what it means to be an “Online Warrior,” including how they mobilize coping strategies, resistance, and relational resources to reframe cyberaggression.

Consistent with the Social Ecology of Resilience Framework, resilience in the context of cyberaggression involves both youth’s internal capacities and the relational resources that support their responses to online challenges. Among these individual and relational factors, empathy has been identified as an important capacity that may shape how youth understand, respond to, and navigate experiences of cyberaggression (Brewer & Kerslake, 2015; Dağcı-Günal & Ayaz-Alkaya, 2025; Liu et al., 2023). Empathy refers to an individual’s ability to experience and understand others’ emotions (Weisz & Cikara, 2021) and is not a fixed trait but a capacity shaped by motivational, attentional, and situational factors (Cheng et al., 2017). Research has distinguished between two dimensions of empathy: cognitive empathy, which involves recognizing and understanding others’ perspectives and emotional states, and affective empathy, which involves sharing or responding to others’ emotional experiences (Cuff et al., 2016). However, the relationship between empathy and cyberaggression remains complex. For example, Falla et al. (2023) found that affective empathy influenced associations among cyberaggression perpetration, cognitive restructuring, and minimization of responsibility, whereas cognitive empathy did not. In contrast, Hinduja and Patchin (2022) found that cognitive empathy was inversely associated with cyberaggression among U.S. youth, while affective empathy was not. A systematic review and meta-analysis further indicated that cyberaggression perpetration was associated with lower levels of both cognitive and affective empathy, although empathy was not significantly related to cybervictimization (Zych et al., 2019). These mixed findings highlight the need to further examine how empathy may function as a protective or adaptive resource within specific cultural and social contexts, particularly among youth experiencing identity-based forms of cyberaggression.

Given the gaps identified regarding coping strategies, resilience, and empathy in cyberaggression research, three research questions guided this study: (1) What kind of experiences did racialized youth have with cyberaggression, and how did racialized youth respond to cyberaggression? (2) How did racialized youth cope with cyberaggression by drawing on the “new warrior” approach? (3) How did a “typical” youth cope with cyberaggression experiences using an empathy map as a culturally relevant method in cyberaggression research?

## 2. Materials and Methods

### 2.1. Study Design

The present study used a concurrent mixed-methods design (J. Creswell et al., 2011; J. W. Creswell & Zhang, 2009), in which quantitative and qualitative data were collected in the same study phase and integrated during interpretation. This design was selected to examine racialized youth’s experiences with cyberaggression from multiple perspectives, including the percentages of reported cyberaggression experiences and behaviors, youth’s self-described coping strategies, and their collective meaning-making regarding responses to cyberaggression. Quantitative data were collected through a self-report questionnaire, whereas qualitative data were collected through open-ended survey questions and a collaborative online empathy mapping activity conducted within a Sharing Circle format. The integration of these complementary data sources allowed for a more contextualized understanding of how racialized youth experienced, interpreted, and responded to cyberaggression.

### 2.2. Participants

Eligibility criteria included youth aged ≤25 years who used digital technologies and communicated in English. Thirteen youth (aged 13 to 25 years) participated in a two-hour online Sharing Circle. Most participants identified as male (*n* = 11) and heterosexual. Participants self-identified as Black (*n* = 12) and Indigenous (*n* = 1). Participants resided in Canada (*n* = 4), the United States (*n* = 4), Kenya (*n* = 4), and South Africa (*n* = 1).

### 2.3. Procedure

Participants were recruited online through social media accounts, particularly Facebook and Instagram. The recruitment posts were boosted paid ads for four weeks in Spring 2022 on Facebook and Instagram. The recruitment ads instructed participants to register by emailing the lab or providing their email address on a Qualtrics survey (www.qualtrics.com; Provo, UT, USA). The recruitment ads included the lab email address and a link to the Qualtrics sign-up survey. Recruitment relied on voluntary participation through online advertisements, which enabled engagement with youth across multiple geographical locations. A member of the research team emailed participants an informed consent form. A research team member contacted participants who returned a signed informed consent form to arrange a date and time in June 2022 to participate in the Sharing Circle. Participants joined a Sharing Circle for two hours via an online video conferencing platform (e.g., Zoom; www.zoom.com; San Jose, CA, USA). At a Sharing Circle, the research team orally reviewed the informed consent form. Participants turned on their videos and audio while the research team reviewed the informed consent form. The research team obtained participants’ permission to video- and audio-record the Sharing Circle. Participants could choose their preferred communication style, including video, audio, and/or chat. Several participants turned off their video while the session was being recorded. Four researchers (one doctoral student, two master’s students, and one faculty member) facilitated the activities during the online Sharing Circle. Research team members took turns leading each activity during the Sharing Circle. The research team explained the purpose of each activity and provided guidance as needed. Participants were informed that all data would be de-identified to protect confidentiality and privacy. For participants under 18 years of age, parental consent and participant assent were obtained prior to participation. This study received ethical approval from the Behavioral Ethics Committee of the University of British Columbia (certification: H21-02364). The online Sharing Circle was semi-structured to facilitate dialogue and collaboration (see Table 1).

After an interactive icebreaker activity, participants completed a short online questionnaire delivered on Qualtrics that consisted of two parts: (i) demographic information and (ii) online experiences and device use. The questionnaire provided descriptive information regarding participants’ demographic characteristics, technology use, and cyberaggression experiences.

To self-identify ethnicity, participants were prompted with “People sometimes identify themselves by the racial or ethnic, or cultural group to which their parents, grandparents or ancestors belong. How do you identify your race or ethnic background?” Respondents were asked to check one response that they most closely identified with: Aboriginal origins (for example, First Nations, Inuit, Métis); African/Caribbean (Black); Asian (Chinese, Korean, Japanese, Taiwanese, etc.); Caucasian (White, European); Latin American (Mexican; South American); Middle Eastern (Arabic, Iranian, Kuwaiti, Persian, Israeli, etc.); South Asian (Indian, Indonesian, Pakistani, Filipino, etc.); Other (tell us); I don’t know.

Participants reported on different aspects of their device use, including an estimate of the amount of time spent communicating and socializing with friends (Shapka & Maghsoudi, 2017). Sample questions include “Do you have access to cellular data?” with a range of responses from “No, I can only access the internet through Wi-Fi” to “Yes, and I never have to worry about my cellular data usage.” Participants were asked “On a typical day, what proportion of the time during which you are able to use your mobile device (such as when you are not sleeping, not restricted by parents, not in class, or not in structured activities) do you spend using your device?” with a 4-point Likert scale from 1 = almost none (less than 25% to 4 = almost all of my time (more than 75%). Participants were asked to reflect on “how much time do you spend using your device compared to your friends/peers?” with a 5-point Likert scale from 1 = much less to 5 = much more. They were asked to indicate how much time they spent on a range of online activities, including social media sites (e.g., Instagram, Snapchat, TikTok, YouTube, Facebook).

Participants’ cyberaggression experiences were measured with the Cyber-Aggression and Cyber-Victimization (CAV) Scale (Shapka & Maghsoudi, 2017). The CAV consists of 24 items with two subscales: cyberaggression perpetration (CAV-P; 12 items) and cybervictimization (CAV-V; 12 items). This behavior-based measure asks respondents about behaviors that constitute cyberaggression and cybervictimization. Items are scored on a 5-point Likert scale ranging from 0 = never to 4 = all the time. The cyberaggression items are grouped together, followed by the set of cybervictimization items. Sample CAV-P items include “Have you posted or re-posted something embarrassing or mean about another person online?” and “Have you posted or re-posted something private about another person that he or she did not want others to know?” Sample CAV-V items include, “Have you had hurtful comments made about an online photo or video of you?” and “Have you received hurtful comments about your perceived sexual behaviors (for example, been called a slut or a pervert)?”. The reliability for the entire measure, as estimated by the Spearman-Brown coefficient for split-half reliability, was α = 0.62 (Shapka & Maghsoudi, 2017). The validity of CAV was explored using a sample of younger youth (aged 11 to 16) in a Canadian context (Shapka & Maghsoudi, 2017). Construct validity was evaluated using confirmatory factor analysis, with α = 0.57, suggesting each subscale measures a specific domain. The correlation between the two factors was 0.57, suggesting that each factor measured a specific domain. Specifically, the factors had alphas of α= 0.83 for CAV-P and 0.90 for CAV-V (Shapka & Maghsoudi, 2017). In addition, two open-text questions (“How have you tried to cope with others being mean online? Please share.” and “What does being an Online Warrior mean to you? Please share.”) captured participants’ subjective interpretations of coping strategies and resilience in response to cyberaggression.

A novel interactive online empathy mapping activity was based on two methods, namely Collective Storytelling and Design Thinking (Sam et al., 2022). An online empathy map looks like an incomplete persona character with specific prompts about online experiences that participants creatively develop into a persona story as a group. The research team introduced Miro (miro.com; San Francisco, CA, USA), an online whiteboard used as a collaborative tool to visually design products. Participants did not need to log on to Miro; instead, the research team used Zoom’s screen-sharing function to display the online empathy map as they entered information based on participants’ discussions. Participants were first instructed to provide demographic characteristics (e.g., name, age, gender, family life) for the persona story and to represent a “typical” youth. The online empathy map was divided into multiple quadrants labeled “How do they spend time online,” “How have they experienced someone being mean online,” “How are they a warrior,” “What do they see,” “What do they say,” “What do they do,” “What do they hear,” “What do they think,” and “What do they feel?” Each quadrant was designed to portray a persona’s sensory experiences. These prompts supported collective reflection and perspective-taking by allowing participants to explore cyberaggression experiences through a shared persona story rather than solely through individual disclosure.

### 2.4. Data Analysis

Descriptive statistics summarized participants’ reported online experiences, device use, and cyberaggression behaviors for each questionnaire item. Given the small sample size, quantitative analyses were limited to descriptive frequencies and percentages. These analyses were not intended to test hypotheses, examine statistical associations, or generate population-level estimates; instead, they provided a descriptive context for participants’ reported online experiences and cyberaggression behaviors. Accordingly, the quantitative component addressed the first research question by characterizing the range and nature of cyberaggression experiences reported by participants. Percentages are presented as descriptive summaries of this sample and are interpreted cautiously, as they are not intended to represent prevalence estimates or generalizable patterns beyond the participants included in this study.

Open-text responses were analyzed using qualitative content analysis. The analysis followed a semantic content analysis approach, with initial coding conducted to remain close to participants’ descriptions and meanings. Rather than applying predefined coping categories, the first phase of analysis involved identifying patterns in participants’ responses about how they responded to cyberaggression and what being an “Online Warrior” meant to them. Codes were developed based on participants’ words and the content of their responses.

Following the initial coding process, codes were reviewed and organized into broader conceptual categories that captured similarities across participants’ responses. At this stage, categories such as avoidance/disengagement coping, distraction coping, and active coping were developed to represent patterns identified in the data. These categories were informed by participants’ descriptions and subsequently interpreted in relation to existing conceptual understandings of coping and resilience. Similarly, responses related to the Online Warrior concept were organized into broader resilience-related characteristics, including personal competence and identity, and endurance, persistence, and advocacy.

Coding was conducted initially by the primary investigator, who completed the first round of coding and developed preliminary categories. The preliminary codes and interpretations were then discussed with graduate research assistants in collaborative Zoom meetings. These discussions provided opportunities to examine alternative interpretations, refine category definitions, and ensure that the final categories remained grounded in participants’ responses. Through this iterative process, the research team reached agreement on the organization and interpretation of the final categories.

To support credibility and trustworthiness, the analysis-maintained reflexivity memos of coding decisions and included collaborative reflection throughout the analytic process. Given the brevity and limited detail of several open-text responses, the analysis did not aim to develop an exhaustive thematic account, but rather to identify patterns in how participants described coping with cyberaggression and the meanings they attributed to the Online Warrior concept.

The collaborative empathy mapping activity was analyzed differently from the open-text responses because it was not meant to generate individual-level themes, but to facilitate collective story construction, interpretation, and meaning-making. During the Sharing Circle, graduate research assistants worked collaboratively with participants to complete the empathy mapping activity and co-construct a persona story representing shared experiences and perspectives related to cyberaggression. Participants’ discussions focused on the persona’s online experiences, emotional responses, coping strategies, challenges, and sources of support.

Consistent with relational and culturally grounded approaches to storing, knowledge generation occurred through collective interpretation and dialogue rather than through reducing narratives into discrete coded categories. Therefore, the empathy mapping activity was not subjected to conventional content coding. Instead, the research team engaged in an interpretive synthesis process, reviewing and refining the collectively generated empathy map through collaborative reflection meetings. The resulting persona was developed as a composite narrative that integrated patterns represented across the empathy map, including online engagement, cyberaggression experiences, emotional impacts, coping responses, and resilience processes.

The persona was not intended to represent any individual participant, but to represent shared experiences across participants. This process served as a knowledge synthesis step, connecting the collaborative empathy mapping activity with the final persona story while preserving the complexity and relational nature of participants’ experiences.

Together, these complementary analytical approaches supported interpretation across multiple data sources. The questionnaire findings characterized participants’ reported cyberaggression experiences (Research Question 1), open-text responses illuminated individual coping strategies and meanings associated with being an Online Warrior (Research Question 2), and the collaborative empathy mapping activity provided a culturally grounded exploration of collective experiences and responses to cyberaggression (Research Question 3).

## 3. Results

Results are presented according to the three research questions guiding this study. First, descriptive questionnaire findings describe participants’ online engagement, involvement in cyberaggression, and experiences of cybervictimization. Second, open-text responses provide insight into how participants described coping with cyberaggression and the meaning they attributed to being an “Online Warrior.” Third, collaborative empathy mapping through the Sharing Circle illustrates how participants collectively represented cyberaggression experiences, emotional responses, and coping strategies by developing a persona story.

### 3.1. Cyberaggression Experiences Among Racialized Youth

Given item-level variability in response completion, descriptive statistics are presented using the number of valid responses for each item. Percentages reflect the proportion of participants who responded to each item; therefore, denominators may vary across items and may not always correspond to the total sample size (*n* = 13). Quantitative analyses were limited to descriptive frequencies to characterize participants’ reported online engagement, device use, and cyberaggression experiences. These findings are presented descriptively and are not intended to estimate prevalence, examine associations, or make population-level inferences.

*Online engagement and device use.* Regarding device use, participants reported a range of activities and online experiences. Most participants (9 out of 13, 69%) reported that they usually have enough data and do not worry about data usage. A total of 77% (10 out of 13) of participants reported spending most of their time on their devices on a typical day. A total of 69% (9 out of 13) of participants perceived they spent somewhat or much more time on their device than their friends and peers. A total of 54% (7 out of 13) of participants spent their time looking at or scrolling through content on social media. A total of 46% (6 out of 13) of participants reported uploading, sharing, and posting content about themselves on social media (for example, live streaming, uploading pictures or videos). A total of 46% (6 out of 13) of participants stated they spent time online watching TV shows or movies. A total of 46% (6 out of 13) of participants reported that all or most of their social media profiles were public. A total of 46% (6 out of 13) of participants had over 1,000 followers on their most popular social media site (e.g., Instagram, Snapchat, TikTok, YouTube, Facebook) and were actively trying to gain more followers. A total of 31% (4 out of 13) of participants stated they often or always valued gaining and maintaining followers. Yet, 38% (5 out of 13) of participants stated that likes, comments, shares, views, and replies on social media were rarely or sometimes important to them.

*Cyberaggression perpetration experiences.* Participants reflected on their experiences with cyberaggression perpetration. A total of 50% (6 out of 12) posted or sent messages to purposely exclude a certain person or group of people at least once a week. A total of 50% (6 out of 12) of participants posted or sent hurtful messages about others’ perceived sexual behaviors (e.g., called somebody a slut or pervert) at least once a week. A total of 42% (5 out of 12) of participants posted, re-posted, or texted an embarrassing photo or video of someone that they did not want others to see at least once a week. A total of 42% (5 out of 12) posted or re-posted something private about another person that they did not want others to know at least once a week. A total of 42% (5 out of 12) used social media to spread rumors or gossip about others at least once a week. A total of 42% (5 out of 12) posted or sent hurtful messages about others’ perceived sexual orientation several times a week. Similarly, 42% (5 out of 12) of participants sent something sexual to others online to embarrass or to be mean to them at least once a week. Participants reported a range of cyberaggressive behaviors.

*Cybervictimization experiences.* Participants considered their experiences with cybervictimization. A total of 67% (8 out of 12) have been purposely excluded online at least once a week. Several times a week, 58% (7 out of 12) of participants had something a person posted or reposted about them online that they did not want others to know. Further, 58% (7 out of 12) of participants had received hurtful comments or messages about their race or ethnicity several times a week. A total of 55% (6 out of 11) of participants have received hurtful comments or messages about their perceived sexual orientation at least once a week. A total of 50% (6 out of 12) of participants had something embarrassing or mean posted or re-posted about them online at least once per week. A total of 50% (6 out of 12) of participants received a hurtful message from others by email, text, or chat at least once a week. A total of 45% (5 out of 11) of participants had sexual content (for example, photos or jokes) sent to them from others who were trying to be mean or embarrass them at least once a week or more. A total of 25% (3 out of 12) of participants reported that hurtful comments about a photo or video of themselves happened every day. Participants experienced cybervictimization through hurtful posts or comments.

### 3.2. Coping Strategies and Online Warrior Characteristics

Participants responded to two open-text questions regarding how they coped with others being mean online and what being an “Online Warrior” meant to them. Direct verbatim quotes from participants are presented in Table 2. Responses were analyzed using qualitative content analysis. Responses were first coded inductively while staying close to participants’ descriptions. They were subsequently organized into broader categories reflecting coping strategies and resilience-related characteristics.

For the first question, “How have you tried to cope with others being mean online? Please share,” three responses were excluded due to insufficient detail (e.g., “no,” “NA,” “yes”), and two responses were missing. Participants described several coping strategies, including talking with the person involved (*n* = 3), ignoring negative interactions (*n* = 2), and taking time away from online spaces (*n* = 2).

Three coping strategy categories were identified: avoidance/disengagement coping, distraction coping, and active coping (Table 2). Avoidance/disengagement coping included ignoring negative interactions and stepping away from online spaces. Distraction coping involved shifting attention toward other activities, such as gaming or watching movies. Active coping included directly communicating with others, attempting to resolve conflicts, educating others about respectful online interactions, and seeking support from peers.

For the second question, “What does being an Online Warrior mean to you? Please share,” one response was excluded due to insufficient detail (e.g., “no”), and one response was missing. Participants described the concept of being an Online Warrior as meaningful (*n* = 2) and helpful (*n* = 2). Participants associated the Online Warrior concept with problem-solving, including attempts to “avert conflict,” determine appropriate responses to harsh comments, maintain online safety, express individuality, and support others.

Two resilience-related characteristics were identified from participants’ descriptions of the Online Warrior concept: personal competence and identity, and endurance, persistence, and advocacy (Table 2). Participants described an Online Warrior as someone who could maintain confidence and authenticity online while navigating challenging experiences. For example, participants described being “brave on social media,” “being safe, but not restricted,” and continuing despite encountering “harsh comments online.” Together, these responses illustrate how participants connected online coping with broader ideas of self-confidence, safety, and positive engagement in digital spaces.

### 3.3. Collective Persona Story Through Empathy Mapping

After analyzing the questionnaire and open-text responses, participants engaged in a collaborative empathy mapping activity during the online Sharing Circle to synthesize and represent their collective understanding of cyberaggression experiences. Rather than generating a new set of qualitative themes, empathy mapping provided a structured process for organizing participants’ shared perspectives regarding what a young person experiencing cyberaggression might think, feel, say, do, hear, and need. The resulting empathy map informed the co-construction of a composite persona story that served as a knowledge translation product reflecting the integrated findings while maintaining participant confidentiality. Verbatim statements generated during the empathy mapping activity are presented to illustrate the collective persona developed by participants.

The collaborative empathy mapping activity conducted through the online Sharing Circle allowed participants to discuss sensitive cyberaggression experiences by constructing a collective persona. Rather than focusing on individual disclosures, participants responded to empathy map prompts by describing what the persona experienced, thought, felt, and did in online spaces. This approach supported discussion of cyberaggression experiences while maintaining confidentiality. Statements are presented verbatim to illustrate the collective persona story participants developed.

**Figure 1:**
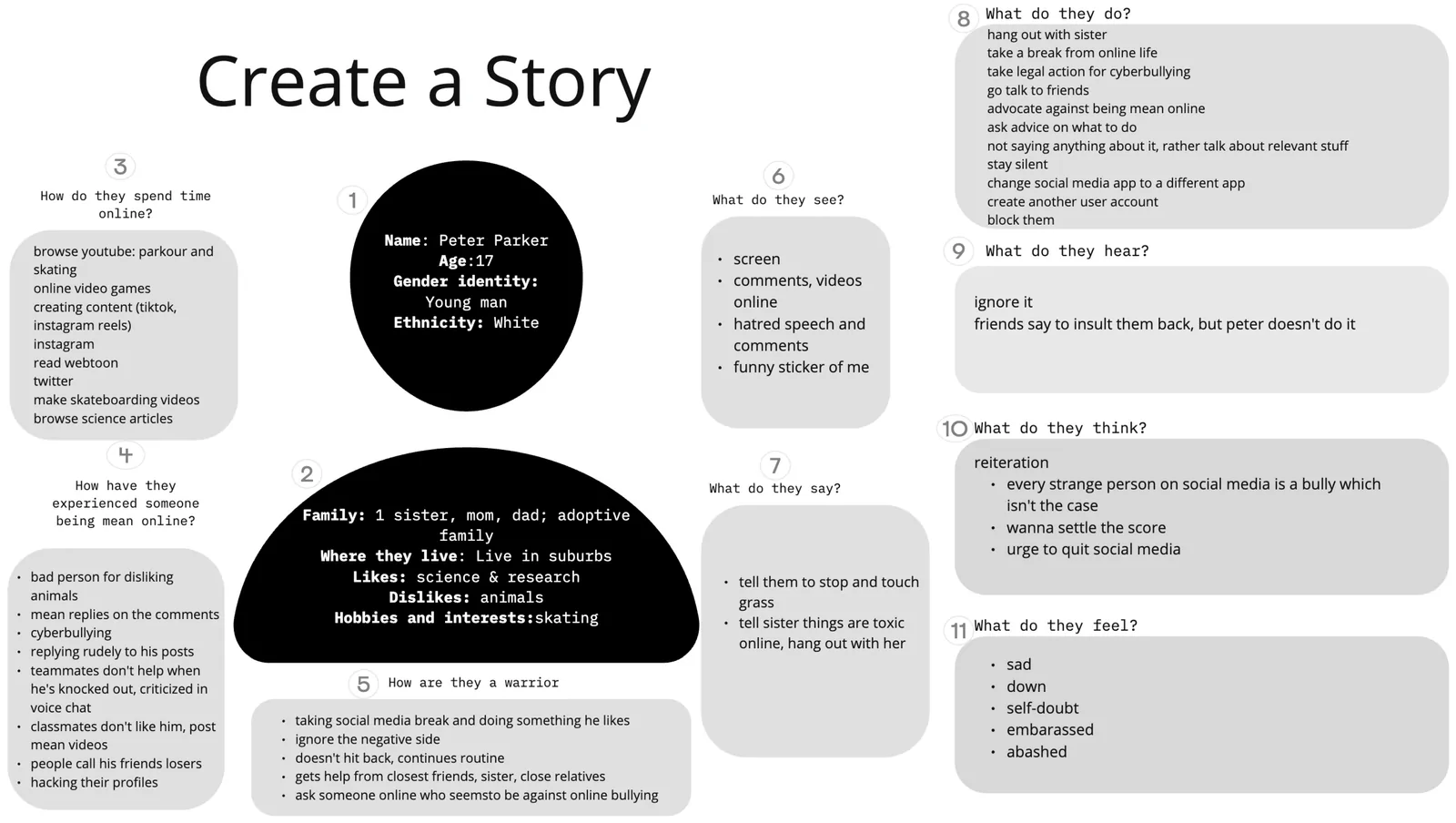
Persona story of Peter Parker.

Participants selected “Peter Parker,” a 17-year-old White male youth, as the composite persona representing their shared understanding of cyberaggression (Figure 1). Rather than reflecting a single participant’s experiences, Peter represented a collective character constructed from participants’ discussions. Participants described Peter as living with his adoptive family in a suburban community and portrayed him as interested in science, skateboarding, gaming, and social media. These characteristics provided a familiar context through which participants could explore sensitive cyberaggression experiences without identifying themselves or others.

Through the empathy mapping process, participants described Peter as actively engaging in multiple online spaces, including YouTube, TikTok, Instagram, Twitter, and online gaming platforms. Participants portrayed him as creating skateboarding content, interacting with friends, watching videos, reading webtoons, and searching for science-related information. At the same time, Peter was represented as experiencing multiple forms of cyberaggression, including hateful comments, harassment during online gaming, targeted messages, account compromise attempts, and witnessing aggression directed toward his friends.

Through the persona story, participants represented Peter as experiencing multiple forms of cyberaggression. These experiences included receiving negative comments related to his interests and identity, experiencing online hate speech, receiving mean replies to his posts, and encountering hostile interactions during online gaming. Participants described situations in which teammates criticized Peter during voice chats, classmates posted hurtful videos or stickers about him, and others attempted to compromise his social media account. Participants also represented Peter as witnessing aggression directed toward others, including negative comments about his friends.

Participants described Peter as feeling sad, embarrassed, frustrated, and increasingly doubtful of himself following harmful online interactions. Although his friends sometimes encouraged retaliation or “settling the score,” participants portrayed Peter as wanting to respond constructively while also questioning whether remaining active online was worthwhile. These discussions reflected the emotional complexity associated with navigating cyberaggression while maintaining friendships and online identities.

Participants described Peter as drawing upon multiple coping strategies when responding to cyberaggression. These included taking breaks from social media, engaging in enjoyable offline activities, maintaining daily routines, ignoring harmful comments, blocking offending users, and seeking support from family and friends. Participants also portrayed Peter using active coping strategies, such as communicating with others, supporting peers experiencing online harm, advocating against bullying, and considering formal reporting mechanisms. Collectively, these responses showed that participants viewed coping as flexible and context-dependent rather than relying on a single strategy.

Overall, the collaborative empathy mapping activity synthesized patterns identified across the questionnaire findings and open-text responses into a single contextualized representation of cyberaggression. The persona illustrated how participants understood the interconnected emotional, behavioral, and relational aspects of online harm, including experiences of victimization, perpetration, bystander involvement, coping, and peer support. Rather than presenting new analytic themes, the empathy map provided an integrated representation of participants’ collective understanding of cyberaggression while preserving individual confidentiality.

### 3.4. Integration of Mixed-Methods Findings

The integration of quantitative and qualitative findings provided a more comprehensive understanding of racialized youth’s experiences with cyberaggression. Across the three data sources, participants’ experiences reflected the complexity of online interactions, including both engaging in cyberaggression and being targeted by it.

The descriptive questionnaire findings established the broader context of participants’ online experiences, showing that participants reported both perpetrating cyberaggression and experiencing cybervictimization. Participants’ open-text responses further elaborated these findings by describing how they responded to harmful online interactions. Specifically, participants identified coping approaches involving avoidance, distraction, and active engagement, while also describing characteristics associated with being an “Online Warrior,” including maintaining identity, seeking safety, and supporting others.

The collaborative empathy mapping activity synthesized patterns identified across both the quantitative and qualitative findings into a single contextualized representation. Rather than generating additional findings, the persona story illustrated how participants understood the relationships among online engagement, cyberaggression experiences, emotional responses, coping strategies, and resilience. This synthesis demonstrated complementarity and expanded the methodological strands adopted for the other two data sources, while preserving the complexity of participants’ experiences.

Overall, integration across the three components of the study provided complementary insights into racialized youth’s experiences with cyberaggression. The questionnaire characterized participants’ online engagement and reported cyberaggression experiences, the open-text responses explained how participants described coping and resilience, and the collaborative empathy mapping activity synthesized these findings into a holistic, practice-oriented representation of youth’s lived experiences. Together, these integrated findings highlighted that cyberaggression involves overlapping experiences of victimization, perpetration, bystander engagement, emotional responses, and adaptive coping within digital environments.

## 4. Discussion

Cyberaggression has long been a ubiquitous problem among youth, transcending cultural and geographic borders (Henares-Montiel et al., 2022; M. F. Wright et al., 2018). To our knowledge, this is the first study to engage mostly young Black men who reported cyberaggression perpetrator behaviors via an online empathy mapping technique through Sharing Circle discussions. We explored their lived online experiences and how they coped with cyberaggression through quantitative and qualitative approaches. The present study findings have been convergently discussed to gain a nuanced understanding of bias-based cyberaggression and coping strategies among racialized youth.

The present study found that some youth experienced bias-based forms of cyberaggression in which their identities or aspects of their lived experiences were targeted (e.g., sexual orientation and gender identities). Past research has explored the lived experiences of Black sexual minority cybervictims (Mereish et al., 2019). In particular, lesbian, gay, and bisexual youth were more likely to experience bias-based cybervictimization (Mereish et al., 2019; Sherwood et al., 2025). Further, the disparities in mental health problems (e.g., suicidal ideation and substance use) were explained to some extent by young sexual minority Black people’s increased risk of bias-based cybervictimization (Mereish et al., 2019). Yet, the present study explored the lived experiences of mostly Black youth who were actively engaged in cyberaggression perpetration and victimization. That is, Black heterosexual young men who, on the one hand, engaged in cyberaggression perpetrator behaviors by targeting others online for their perceived sexual orientation and gender identities; on the other hand, they experienced cybervictimization as hurtful comments or posts about their race or ethnicity. The possible interpretations of the findings are that social media sites offer racialized youth a wide audience on public platforms, which might explain why they felt freer to act on their biases and discriminatory attitudes to target individuals with minority identities. In other words, online disinhibition might play a role in perpetrating bias-based cyberaggression. These interpretations were not directly tested in the present study; thus, until corresponding findings are known, they remain tentative. Our results are noteworthy because there is limited past research revealing that racialized youth can experience bias-based cybervictimization (Polanco-Roman et al., 2025; Tynes et al., 2024, 2025) and a paucity of studies on their cyberaggression behaviors. The present study captures their cyberaggression experiences in both breadth (quantitative data) and depth (qualitative open-text data).

The present study findings revealed that racialized youth will seek social support as a coping strategy. Specifically, peers, close relatives, and siblings were identified as social support to help cope with cyberaggression. Although the result was based on a small sample size and should be interpreted with caution, it is consistent with previous international findings indicating that youth relied more on social support seeking for public cybervictimization (M. F. Wright et al., 2018). Another consistent finding was retaliation as a coping strategy (M. F. Wright et al., 2018), and the present persona story suggests that youth may retaliate. These qualitative findings should be considered alongside quantitative findings indicating the use of public social media accounts and the active pursuit of followers. This may increase racialized youth’s feelings of embarrassment, which could result in seeking online retaliation illustrated in the persona story. This may increase the risk of youth wanting to “settle the score” in response to public cyberaggression posts and comments, which may be connected to negative emotions. Present study findings indicate that racialized youth took an active coping strategy approach to respond to cyberaggression through open communication and education to help themselves and others. This could suggest that they may have strong connectedness to school or prosocial behaviors in daily life (Chan & Wong, 2017), which may make them more likely to use problem-solving coping strategies and reduce the negative impact of cyberaggression. Relatedly, resilience was exemplified in their stress coping ability in response to cyberaggression. The present study findings revealed two factors identified in the resilience literature: (1) the notion of personal competency and identity; and (2) stress endurance, high standards, and tenacity (Connor & Davidson, 2003). A better understanding of resilience and coping strategies may help educators and researchers develop more tailored interventions to prevent cyberaggression among diverse youth. For example, prevention and intervention programs can focus on fostering a positive environment that supports youth in using problem-solving coping strategies and building resilience when they encounter cyberaggression (Ungar, 2011).

Scholars advocate for changes in cyberaggression research to better capture the complexities of cyberaggression among youth (Kofoed & Staksrud, 2019). For instance, researchers have engaged youth in creating graphic vignettes about cyberaggression to provide insight into how cyberaggression situations emerge, how cyberaggression influences their mental health, the coping strategies they use, and how cyberaggression is experienced (Khanolainen & Semenova, 2023). Researchers rarely offer racialized youth an opportunity to share their lived cyberaggression experiences in their own voices. The present study provided racialized youth who self-reported cyberaggression perpetrator behaviors with an opportunity to discuss their cyberaggression experiences in a Sharing Circle via co-creating a persona story using an online empathy map; however, their persona story did not fit the overall cyberaggression perpetrator narrative. They did not present the persona story as a cyberaggression perpetrator; instead, they co-created a persona story about a young man being cyberbullied themselves. Interestingly, although most participants are Black youth, they described a White youth as the main character, which might reflect the dominant White narrative culture. This possible interpretation was not examined in the present study; thus, its potential impact on cyberaggression warrants deeper consideration in future research. These findings suggest that creative activities may help racialized youth connect with and articulate their feelings and emotions.

Indeed, different components of the persona story amplified a cybervictimization perspective and conveyed empathetic attitudes. In the current study, cyberaggression experiences were associated with a range of negative emotions, including feelings of sadness, self-doubt, embarrassment, and abashment. The persona story also demonstrated how racialized youth viewed others around them (online, family, friends) to better understand coping strategies important for reducing cyberaggression. Given that quantitative studies rely on youth’s honest self-report of experiences, our study has important implications for better understanding the unique perspectives of racialized youth engaged in cyberaggression perpetration. With empathy being a predictor (Hinduja & Patchin, 2022; Zych et al., 2019) and outcome (Falla et al., 2023) of cyberaggression perpetration, we recommend using flexible online empathy mapping to co-create persona stories to study cyberaggression and capture novel, multifaceted dimensions.

Overall, the quantitative results presented descriptive and exploratory findings on the general online experiences and cyberaggression behaviors of the present sample. While it offered insights into a broad range of experiences and behaviors, relying solely on the quantitative results can lead to a lack of in-depth examination (which is typical of quantitative results) of how youth cope with cyberaggression and what coping means to them. This was complemented by qualitative results from open-text responses, which can reveal how participants cope with cyberaggression and what being an Online Warrior means to them. The questionnaire and open-text findings addressed research questions 1 and 2. However, both the quantitative and qualitative results focused on a general methodological approach, which may overlook the specific perspective racialized youth can take; thus, results from a culturally relevant approach, “empathy mapping”, were also presented to complement previous results and answer our research question 3. By presenting the findings from the three different data sources, the present study provided unique insights into cyberaggression experiences among racialized youth from a mixed-methods perspective.

While these findings contribute to the literature, they should be considered within the study’s limitations. First, due to convenience sampling and a small sample size (*n* = 13), the participants in the present study were not representative of racialized youth populations, particularly girls, given the gender imbalance (most participants were boys). However, the present study primarily focused on the interpretation of the qualitative aspect, where a small sample size is typical and acceptable. Second, the online recruitment method may introduce self-selection bias, whereby youth with strong opinions and proficiency in English may join the study, leaving other youth underrepresented. Third, because participants were from different countries, cross-national heterogeneity could limit the generalizability of the interpretation and lead to semantic saturation. However, the present participants may represent diverse sociocultural contexts, which may reflect differences in their online behaviors and coping strategies. This diversity may provide unique insights for researchers and practitioners globally. Fourth, our data rely on self-report, which could lead to social desirability bias, in which youth’s responses align with social expectations and potentially mask the true patterns in the data. However, social desirability bias may be reduced by the empathy mapping method adopted in our study. It is a trauma-informed approach based on socio-emotional learning. It draws on personal and peer experiences rather than solely on self-reported quantitative data, which also makes our methods innovative. Lastly, the single sharing circle may provide a relatively narrow scope of cyberaggression experiences. However, the present sample primarily consists of cyberaggression perpetrators, which is special and makes their data unique. The dual role (i.e., cyberaggression perpetration and victimization) of “Peter Parker” created by the circle also complemented the quantitative findings well and offered in-depth interpretation. Future work should consider longitudinal designs for self-administered questionnaires to provide a richer understanding of bias-based cyberaggression and insight into the role of coping strategies in response to cyberaggression. Considering the present study findings, it explored bias-based cyberaggression experiences, which coping strategies were adopted, and what emotional experiences they had among racialized youth. These descriptive findings may inform inclusive prevention and intervention strategies for diverse youth. Despite these limitations, the study’s strength should be acknowledged. In particular, as far as we know, this is the first study addressing cyberaggression and coping strategies among racialized youth via an online empathy mapping technique.

## 5. Conclusions

We live in a diverse and increasingly connected global society. Studying bias-based cyberaggression is important for supporting diverse youth in borderless online spaces. The present study filled a gap by identifying coping strategies and cyberaggression experiences of racialized youth. Combining quantitative and qualitative findings provides a fuller picture of cyberaggression. This approach examined device use and online experiences without a deficit lens, which is a step towards supporting more inclusive and culturally responsive research practices for racialized youth. The implications of this work may inform future inclusive and diverse interventions and prevention strategies for young Black men who may be engaged in cyberaggression behaviors. As youth form their ethnicity and other identities, such as gender and sexuality, educators, practitioners, and scholars may consider how to develop and implement intervention and prevention efforts that account for the intersectionality of social identities when understanding youth’s online experiences. Given these implications, it is still important to note that this study draws on a convenience sample of youth; therefore, testing the practical implications in a larger population would be crucial for future studies. This will provide a better understanding of how specific coping strategies and bias-based cyberaggression are associated, to deliver continued support for mental health among racialized youth. Given the daily online lives of diverse youth, future research and prevention efforts are needed to develop digital interventions that help those affected by bias-based cyberaggression cope with these experiences.

## Figures and Tables

**Table 1:** Sharing circle activities.

Topic Areas	Activities	Time
Opening Sharing Circle	Participant informed consent	30 min
(Zoom waiting room enabled)	Research team introductions Respectful Sharing Circle protocols	
	Introduction ice-breaker group activity	
Participatory design activity	Online demographic questionnaire	40 min
	Create persona	
Break		15 min
Mobile app design	Demonstrate Qualtrics survey app	25 min
	Discussion on youth’s preferences on a survey app in data collection	
Break		5 min
Closing	Thank you	5 min

**Table 2:** Coping strategies and Online Warrior characteristics identified from open-text responses.

Topic	Category	Direct Text Response Quotes
**Coping strategy**	*Avoidance coping*	By ignoring them
	*Distraction coping*	By taking an online break Gaming Focusing on movies, gaming
	*Active coping*	I just try to talk to them I usually just end up saying they are missing out talking to me and telling other friends about it (not usually ones who know said person) I just educate them on the importance of being helpful online as one can save life. A discussion on interests
**No cyberaggression experiences**	*No cyberaggression*	Nobody has been mean to me online. No idea
**Resilience**	*Notion of personal competence and identity*	Being Brave on social media just being me Being safe, but not restricted It means being a person who can benefit and build a better version of himself online
	*Endurance, high standards and tenacity*	Surviving the harsh comments online Online Warriors sounds like actively trying to avert conflict and standing up against harassment and targeted comments Means a lot It means a lot to me. It is important and keeps me going Really helpful People who help others

## Data Availability

The data presented in this study are available on request from the corresponding author due to privacy concerns.
